# Focal therapy in prostate cancer and advances in the use of androgen blockade

**DOI:** 10.3389/fruro.2025.1599763

**Published:** 2025-10-30

**Authors:** Carlos Hernandez, Juan Ignacio Martinez-Salamanca, Giuseppe Maiolino, Bernardino Miñana, Francisco Gómez-Veiga

**Affiliations:** ^1^ Servicio de Urología, Hospital General Universitario Gregorio Marañón, Madrid, Spain; ^2^ Lyx Institute, Universidad Francisco de Vitoria, Madrid, Spain; ^3^ Dpt. Urology, University Clinic of Navarra, Pamplona, Spain; ^4^ Dpt. Urology, A Coruña University Hospital Complex (CHUAC), A Coruña, Spain

**Keywords:** prostate cancer, focal therapy, magnetic resonance imaging, ablative therapies, androgen deprivation

## Abstract

Focal therapy (FT) for localized prostate cancer (PCa) has evolved into a precision-based alternative to radical treatments, aiming to eradicate clinically significant disease while minimizing functional morbidity. This review provides an updated and critical synthesis of the current landscape of FT, emphasizing the central role of multiparametric magnetic resonance imaging (mpMRI) and fusion biopsy in patient selection, treatment planning, and post-therapy evaluation. mpMRI enables accurate lesion characterization, identification of index lesions, and tailored ablation with energy sources such as high-intensity focused ultrasound (HIFU), cryotherapy, irreversible electroporation (IRE), vascular-targeted photodynamic therapy (VTP), and interstitial laser therapy (ILT). Across modalities, continence preservation exceeds 90%, and erectile function is maintained in up to 100% of patients, underscoring the functional safety of FT. While current evidence supports FT as an oncologically sound option for low- and select intermediate-risk disease, data from phase III trials remain scarce. Emerging strategies integrating androgen deprivation therapy (ADT) with FT show promise in enhancing tumor control, particularly in high-risk, large-volume, or anatomically complex cases. Preliminary studies suggest synergistic benefits without increasing toxicity, though definitive long-term evidence is lacking. This review highlights how the convergence of advanced imaging, ablative technology, and systemic modulation may redefine the therapeutic paradigm of localized PCa. Further prospective, comparative trials are essential to establish the optimal combination strategies, refine patient selection, and confirm durable oncological and functional outcomes.

## Introduction

The widespread adoption of multiparametric magnetic resonance imaging (mpMRI) in most urology departments, together with the development of fusion biopsy (FB) techniques, has led to a growing number of early-stage prostate cancer (PCa) diagnoses—often at less aggressive stages. As observed in other solid organ tumors (kidney, breast, liver,…), focal treatment of localized lesions has become an established approach.

**Figure 1:**
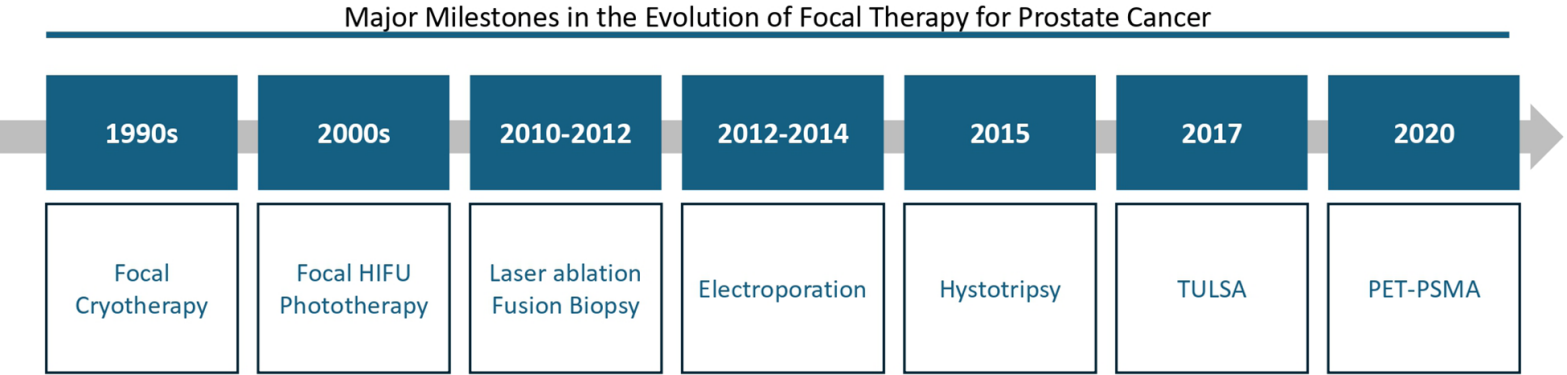
Evolution of focal therapy modalities in prostate cancer: key milestones from 1990 to 2020.

However, due to the multifocal nature of PCa, the clinical implementation of focal therapy (FT) has progressed more slowly. The increasing recognition of the index lesion—the largest and most aggressive tumor focus—as the key driver of PCa progression has expanded the indications for FT (1) (**Figure 1**).

Advances in technology have equipped urologists with highly precise and safe tissue- destructive techniques, which minimize side effects. These include cryotherapy, high-intensity focused ultrasound (HIFU), irreversible electroporation (IRE), radiofrequency ablation, and laser therapy.

Since the 1990s, focal therapy for prostate cancer has advanced through progressively sophisticated strategies. Initially, focal cryotherapy offered a minimally invasive option, followed in the 2000s by high-intensity focused ultrasound (HIFU) and phototherapy for precise, lower-morbidity treatment. Between 2010 and 2014, techniques such as laser ablation guided by fusion biopsy and irreversible electroporation enhanced lesion targeting and non- thermal ablation. Histotripsy emerged in 2015, enabling ultrasound-based tumor destruction without heat, while the 2017 TULSA system integrated MRI-guided transurethral ablation with intraoperative control. Most recently, PSMA PET (2020) has refined lesion detection and post- treatment monitoring, underscoring the critical role of molecular imaging in focal therapy strategies.

Level I scientific evidence has already demonstrated that combining radiotherapy for intermediate- and high-risk localized PCa with androgen deprivation therapy (ADT) for at least six months significantly improves cancer-specific survival (2).

Given the growing role of FT and the aim to enhance cancer-specific survival without increasing toxicity, there is rising interest in investigating whether combining FT with ADT could improve oncologic outcomes—mirroring the established benefit seen with radiotherapy.

This review summarizes current indications for FT based on mpMRI findings and FB results, describes the different energy modalities currently available for prostate tissue ablation, and reviews published and ongoing clinical trials exploring the potential of ADT as an adjunct to FT for prostate cancer tissue destruction.

## Evidence acquisition

This narrative review was conducted in June 2024 using the PubMed database with the following search terms: “prostate cancer,” “focal therapy,” “magnetic resonance imaging,” “ablative therapies,” and “androgen deprivation.”

Human studies published in English or Spanish between January 2010 and June 2024 were included, with no additional restrictions applied.

## Importance of multiparametric magnetic resonance imaging in identifying candidates for focal therapy

Just as the introduction of PSA testing marked a distinct era in PCa management, the integration of MRI is likely to define a new pre- and post-mpMRI era (3). The combination of mpMRI with image-guided FB has revolutionized PCa diagnostics, enhancing detection rates, enabling precise local tumor staging, improving follow-up for patients under active surveillance (4), and assessing local recurrence after FT (5).

### Diagnostic precision and risk stratification

mpMRI improves the detection of clinically significant PCa and reduces the misclassification of high-risk disease as intermediate-risk. Accurate risk stratification is therefore essential to identify candidates suitable for FT (6). Meta-analyses have shown that combining mpMRI with systematic and targeted biopsies lowers upgrading rates from 42% to 27% compared with systematic biopsy alone (7).

### Anatomical localization and lesion characterization

mpMRI accurately assesses the number, size, and location of lesions, correlating closely with histopathology. Turkbey et al. reported positive predictive values for 3-T mpMRI of 98% (overall prostate), 98% (peripheral zone), and 100% (central zone) (6). Villers et al. confirmed similar results with 1.5-T mpMRI, demonstrating robust sensitivity and specificity (8).

This imaging precision enables targeted treatment using transrectal ultrasound-MRI fusion or in-bore MRI guidance, applying energies such as HIFU, cryotherapy, irreversible electroporation (IRE), or laser ablation, while sparing critical structures like the rectum, urinary sphincter, and neurovascular bundles (9, 10).

### Multifocality and targeting index lesions

Most PCa cases are multifocal, which poses a challenge for FT. Current strategies focus on index lesions, targeting all clinically significant ISUP ≥2 lesions while leaving ISUP 1 lesions under active surveillance (6).

### Extent of ablation and safety margins

mpMRI guides the required extent of ablation. Earlier recommendations suggested a 5–9 mm margin beyond the MRI-visible lesion (11). Recent evidence indicates that extending the ablation zone by 10mm beyond the visible lesion captures most clinically significant cancer foci, emphasizing the value of perilesional biopsies during fusion-guided biopsy (11).

Additionally, mpMRI ensures adequate safety margins from critical structures, recommending at least 5mm from the rectum and urinary sphincter (4).

### Anatomical location and modality selection

The “A La Carte” model by the European Section of Urotechnology (ESUT) guides FT modality selection: posterior lesions are best treated with HIFU, anterior lesions with cryotherapy, and apical lesions with focal brachytherapy, minimizing urethral morbidity (12).

### Post-treatment monitoring

MpMRI is essential for follow-up after FT. PI-RADS remains useful for untreated areas, while specialized systems like Prostate Imaging after Focal Ablation (PI-FAB) (13) and a recently developed three-category scoring system (14) evaluate treated zones.

## Types of energy in focal therapy for prostate cancer

Focal therapy for PCa encompasses a range of energy modalities designed to ablate cancerous tissue while preserving surrounding functional structures. The most extensively evaluated techniques include HIFU, cryotherapy, IRE, vascular-targeted photodynamic therapy (VTP), and interstitial laser therapy (ILT). Less commonly used approaches include radiofrequency or microwave ablation. While high- and low-dose-rate brachytherapy can be applied focally, these have been excluded here because they rely on radiation-induced DNA damage rather than direct tissue ablation via thermal, electrical, or photodynamic mechanisms, making them non-ablative therapies.

Currently, no direct comparative studies have systematically assessed the efficacy or safety profiles between these energy modalities, highlighting the need for prospective trials (15). Except for HIFU, most techniques are applied via the transperineal route under TRUS guidance, with careful preservation of critical structures such as the urethra, neurovascular bundles, sphincter, and rectum. Most modalities have been evaluated in IDEAL stages 2a, 2b, and 3, reflecting early development, refinement, or case series experiences (15). Reported functional outcomes are generally favorable, with continence preservation rates above 90% and erectile function maintained in 70–100% of patients, with the highest rates observed for non-thermal mechanisms (15, 16) (**Tables 1**, **2**).

**Table 1:** Comprehensive overview of prostate MRI performance, focal therapy planning, and post-treatment assessment.

Category	Subcategor y/factor	Parameter	Assessment/method	Relevance/advantage	Area of application	Reference
Imaging Performance	Sensitivity/Specificity	Whole prostate	MRI 3 T/1.5 T	High correlation with histopathology/Confirms results	Detection	(6, 8)
	Sensitivity/Specificity	Peripheral zone	MRI 3 T	–	Detection	(6)
	Sensitivity/Specificity	Central zone	MRI 3 T	–	Detection	(6)
mpMRI Evaluation	Number and size of lesions	Lesion volume, ROI visualization	mpMRI	Determines ablation extent	FT planning	(6, 8)
	Multifocality	Detection of additional lesions	mpMRI	Guides whether only index lesions treated	FT planning	(6)
	Ablation extent & safety margin	Distance to critical structures	mpMRI	Prevents rectal/sphincte r injury	FT planning	(4, 11)
	Anatomical location	Peripheral/Anterior/Apical	mpMRI	Guides selection of FT modality	FT planning	(12)
FT Modality	Preferred modality by location	Posterior	HIFU	Minimizes urethral morbidity	FT execution	(12)
	Preferred modality by location	Anterior	Cryotherapy	Precise ablation	FT execution	(12)
	Preferred modality by location	Apical	Focal brachytherap y	Low urethral injury risk	FT execution	(12)
Post-FT Assessment	System	PI-RADS	MRI	Detection of new csPCa	Untreated lesions	(13)
	System	PI-FAB	MRI	Monitoring ablation zone	Treated lesions	(13)
	System	Three- category system	MRI	Most recent post-FT evaluation	Treated lesions	(14)

MRI, Magnetic Resonance Imaging; FT, Focal Therapy; ROI, Region of Interest; csPCa, clinically significant prostate cancer. Sensitivity and specificity values are reported per referenced studies. mpMRI assessments guide lesion characterization, treatment planning, and modality selection. Post-FT evaluation systems monitor treatment efficacy and detect new or residual disease.

**Table 2:** Focal therapy modalities for prostate cancer: mechanisms, recurrence, functional outcomes, and IDEAL stages.

Modality	Mechanism	Treated area recurrence	Untreated area recurrence	Functional preservation	IDEAL stage	Notes
HIFU	Thermal ablation + cavitation	21–35%	18–33%	Continence >90%, Erectile 70– 100%	3	Non-invasive; most clinical experience; mpMRI improves targeting
Cryotherapy	Freeze-thaw cycles to –40°C	~10%	Not always reported	Continence >90%, Erectile 70– 100%	2b–3	Established; imaging guidance optimizes probe placement
IRE	Non-thermal apoptosis via electrical pulses	84–90%	7–33%	Continence >90%, Erectile 70– 100%	2b	Ideal for small/moderate tumors near critical structures; MRI/US fusion essential
VTP	Photodynamic reaction	6–25%	25–31%	Continence >90%, Erectile 70– 100%	2a– 2b	Requires photosensitizer; mpMRI or imaging biomarkers guide delivery
ILT	Thermal ablation via 980 nm laser	20–46%	Not always reported	Continence >90%, Erectile 70– 100%	2a– 2b	MRI-guided; precise targeting for small, well- defined lesions

Recurrence rates reflect mid-term outcomes reported in the literature. Functional preservation is continence and erectile function.

IDEAL stages indicate development level: 2a = early development (red), 2b = refinement (yellow), 3 = assessment in prospective trials (green).

HIFU, High-Intensity Focused Ultrasound; IRE, Irreversible Electroporation; VTP, Vascular-Targeted Photodynamic Therapy; ILT, Interstitial Laser Therapy. Recurrence rates are reported for treated and untreated prostate areas. Functional preservation reflects continence and erectile function outcomes. IDEAL stages indicate the developmental and clinical evaluation phase of each therapy. mpMRI and imaging guidance enhance targeting accuracy and treatment efficacy.

### High-intensity focused ultrasound

HIFU is the only non-invasive modality currently applied for focal prostate therapy. Using spherical transducers (transrectal or transurethral), high-intensity ultrasound beams are focused to generate temperatures of 60–90°C, causing tissue ablation via coagulative necrosis. Mechanical damage from cavitation further contributes to cellular destruction. HIFU benefits from the largest clinical experience, with mid-term treatment failure rates of 21–35% in treated areas and 18–33% in untreated regions (15, 16).

Advantages: Non-invasive approach, precise targeting, and well-documented safety profile.
Limitations: Limited long-term oncological data; efficacy may be influenced by tumor size and location, particularly near the apex or anterior prostate.

### Cryotherapy

Cryotherapy induces tissue destruction through freezing to temperatures as low as –40°C, typically via two freeze-thaw cycles using argon and helium delivered through 17G cryoprobes. Cellular damage results from protein denaturation, osmotic dehydration, and metabolic failure, complemented by delayed vascular injury, which is critical to its effect. Clinical experience is extensive, but oncological control data remain limited, with ablation success around 90% (15, 16).

Advantages: Established technique with long-term functional preservation data.
Limitations: Limited high-level evidence for local cancer control; risk of urethral or rectal injury if critical structures are not carefully avoided.

### Irreversible electroporation

Irreversible electroporation (IRE) delivers high-voltage, low-energy electrical pulses via transperineal electrodes, inducing apoptosis while sparing connective tissue and critical structures. Mid-term tumor control ranges from 84–90%, with 7–33% recurrence in untreated areas. Optimal candidates are patients with localized, small-to-moderate volume tumors near critical structures such as the neurovascular bundles, urethra, or rectum, where thermal ablation carries higher functional risk. Patients with preserved baseline urinary and sexual function benefit most from IRE’s functional preservation. Precise electrode placement, guided by MRI or ultrasound fusion, is essential, and long-term oncological data remain limited (17, 18).

Advantages: Non-thermal mechanism allows safe treatment near sensitive structures; favorable functional outcomes.
Limitations: Limited long-term oncological data; procedure requires precise electrode placement, often guided by MRI or ultrasound fusion.

### Vascular-targeted photodynamic therapy

Optimizing outcomes in focal therapy relies heavily on precise lesion localization and treatment planning using advanced imaging. Multiparametric MRI and emerging imaging biomarkers enable accurate delineation of tumor margins, guide energy delivery, and improve coverage of the target while sparing critical structures. Studies indicate that integrating mpMRI into procedure planning enhances tumor control and maximizes functional preservation, particularly for modalities such as VTP and HIFU, where treatment precision directly impacts both oncological and functional outcomes.

VTP uses an infrared laser fiber and intravenously administered photosensitizer in oxygen-rich conditions to generate reactive oxygen species, inducing apoptosis and vascular damage.

Approved photosensitizers include WST09 (padoporfin) and WST11 (padeliporfina/TOOKAD^®^). Recurrence rates are reported at 6–25% in treated areas and 25–31% in untreated regions (15, 16).

Advantages: Targeted, minimally invasive approach with preservation of surrounding tissue.
Limitations: Requires photosensitizer administration and light delivery expertise; limited long- term outcome data; patient selection is critical.

### Interstitial laser therapy

Interstitial laser therapy (ILT) delivers thermal energy under MRI thermometry guidance to induce precise coagulative necrosis (980 nm diode lasers inserted transperineally or transrectally to deliver thermal energy). Treated area recurrence ranges from 20–46%, slightly higher than HIFU and comparable to cryotherapy, while functional preservation remains consistently high, with continence over 90% and erectile function 70–100%. ILT is particularly advantageous for small, well-defined tumors in anatomically challenging locations, offering precise energy delivery and minimal collateral damage. Although long-term oncological outcomes are limited, careful patient selection and imaging-guided planning optimize both efficacy and preservation of urinary and sexual function (19).

Advantages: Precise thermal ablation with imaging guidance; applicable for localized lesions.
Limitations: Limited long-term oncological evidence; requires specialized equipment and technical expertise.

### Summary and future perspectives

Each energy modality offers unique advantages and challenges, with non-thermal techniques generally providing superior preservation of function near critical structures. Direct comparative trials are lacking, and most studies report mid-term outcomes. Future research should focus on:

- Head-to-head comparisons of efficacy and safety across modalities.
- Optimization of patient selection criteria using imaging and molecular biomarkers.
- Integration with adjuvant therapies, including androgen deprivation or immunomodulatory approaches.

## Focal therapy and androgen deprivation

Focal therapy in PCa is justified by its potential to reduce physical and psychological morbidity while limiting disease progression (20, 21). Optimal patient selection remains crucial, as variability in age, comorbidities, tumor type, and diagnostic approach complicates cross-study comparisons. Most published data are retrospective, phase II, single-arm studies, with only one phase III trial identified (16, 22).

A prospective randomized study comparing FT with active surveillance (AS) in low-risk patients using photodynamic therapy demonstrated that, after four years, disease progression occurred in 28% of FT patients versus 58% in the AS group, with a lower need for salvage treatment (6% vs. 29%) and no significant differences in adverse effects (22). [Improvement comment: Integrated FT vs AS comparison in a continuous narrative for clarity.] ADT was not administered in this cohort. Most studies continue to focus on ISUP grades 1–3 without systematic evaluation of ADT, highlighting a current gap in evidence.

There is a theoretical rationale for combining FT with ADT. Tissue injury from FT, particularly in the peripheral zone, may increase microcirculation density, and transient ADT could reduce vascularity, potentially decreasing recurrence risk (23). The ENHANCE study demonstrated that HIFU combined with ADT improved oncological outcomes without increasing toxicity (13), providing early support for this approach.

Registry studies further illustrate FT outcomes across risk categories. In a cohort including high-risk (32%) and T2b–T3 patients treated primarily with HIFU, five-year follow-up showed metastasis-free survival in all patients, cancer-specific survival of 98–100%, and 98% maintained full continence (24). Similarly, a comparison of 12,433 radical prostatectomies with 442 patients undergoing laser ablation for low-risk disease revealed no significant differences in cancer-specific mortality over 60 months, though ADT use was not clearly defined (25).

These data emphasize the importance of integrating patient selection, tumor characteristics, and procedural precision to optimize outcomes.

FT has also been applied as salvage therapy following progression post-FT or radiotherapy, using cryotherapy, brachytherapy, or HIFU. In these scenarios, ADT has often been included, achieving acceptable biochemical control and low incidence of grade ≥3 genitourinary toxicities (26–28). Imaging guidance, particularly mpMRI, enhances lesion targeting, reduces functional compromise, and may further improve outcomes, especially when combined with ADT.

Current clinical trials remain predominantly phase II, focusing on low-risk populations without ADT, with only six phase II–III trials actively recruiting, four investigating HIFU and one evaluating electroporation (6, 16). Evidence suggests FT is a viable alternative for localized prostate cancer, particularly for low-risk and select intermediate-risk (ISUP 2) tumors (16, 20, 29).

The role of ADT in FT, either to enhance oncological control or expand indications, remains investigational. ADT may reduce prostate volume by approximately 30% and improve urinary symptoms, potentially broadening the candidate pool for FT (30, 31). Combining FT and ADT is particularly relevant for patients with large prostates, significant lower urinary tract symptoms, or high-risk features, although definitive evidence is lacking.

In summary, FT offers favorable functional outcomes and may reduce disease progression compared to AS. The combination with ADT is promising, particularly in high-risk, salvage, or gland-enlarged patients, but high-quality trials are essential to define optimal patient selection, treatment intensification, follow-up strategies, and the precise role of ADT in focal therapy.

## Conclusions

Multiparametric MRI combined with fusion biopsy is essential for accurate patient selection, precise targeting, and effective follow-up in focal therapy for prostate cancer. A range of energy modalities demonstrates comparable ablative efficacy and functional preservation, although direct comparative studies are lacking.

The integration of androgen deprivation therapy (ADT) with focal techniques represents a promising strategy to expand treatment indications, particularly for higher-risk or anatomically challenging cases. Well-designed prospective trials are urgently needed to define optimal patient selection, evaluate combination strategies, and establish long-term oncological and functional outcomes.
